# The role of IL-6, ferritin, and coagulopathy in Covid-19 clinical progression

**DOI:** 10.12688/f1000research.125115.2

**Published:** 2023-09-22

**Authors:** Alvin Tagor Harahap, Cosphiadi Irawan, Adityo Susilo, Kuntjoro Harimurti, Dewi Gathmyr, Hamzah Shatri, Anna Mira Lubis, Leonard Nainggolan, Murdani Abdullah

**Affiliations:** 1 Department of Internal Medicine, Pertamina Central Hospital, Jakarta, 12120, Indonesia; 2 Hematology and Medical Oncology Division, Department of Internal Medicine, Faculty of Medicine, University of Indonesia, Jakarta, 10430, Indonesia; 3 Tropical and Infectious Diseases Division, Department of Internal Medicine, Faculty of Medicine, University of Indonesia, Jakarta, 10430, Indonesia; 4 Department of Internal Medicine, Faculty of Medicine, University of Indonesia, Jakarta, 10430, Indonesia

**Keywords:** D-dimer, Ferritin, Fibrinogen, IL-6, Prothrombin Time

## Abstract

Background

In COVID-19, the release of pro-inflammatory mediators in the cytokine storm, primarily interleukin-6 (IL-6), has been hypothesized to induce pulmonary intravascular coagulation. However, the relationship between IL-6 and coagulopathy remains unclear in COVID-19 progression. We aimed to investigate the correlation of IL-6 with D-dimer, fibrinogen, prothrombin time (PT), and ferritin. Furthermore, we also analyzed the effect of those parameters on the worsening of COVID-19 patients.

Methods

A prospective cohort study was conducted in moderate and severe COVID-19 patients from June 2020 to January 2021. A serial evaluation of IL-6, D-dimer, fibrinogen, ferritin, and PT was performed and correlated with the patient's condition at admission and on the 14th day. The outcomes (improvement, worsening, or discharged patients) were recorded during the study.

Results

Of 374 patients, 73 study subjects (61 severe and 12 moderate COVID-19) were included in this study. A total of 35 out of 61 severe and one out of 12 moderate illness subjects had experienced worsening. Spearman-rank correlation of IL-6 with with ferritin, D-dimer, fibrinogen, and PT was 0.08 ( *p*=0.5), −0.13 ( *p*=0.27), 0.01 ( *p*=0.91), and 0.03 ( *p*=0.77), respectively. In ROC analysis, D-dimer (74,77%) and IL-6 (71,32%) were the highest among other variables (>60%).

Conclusions

In COVID-19 patients, there was a correlation between elevated IL-6 and D-dimer levels with disease deterioration. There was no correlation between elevated IL-6 levels with ferritin, D-dimer, fibrinogen, and PT levels. Therefore, changes in IL-6 and D-dimer can predict worsening in moderate and severe COVID-19 patients.

## Introduction

The SARS-CoV-2 virus is a β-coronavirus with an enveloped, positive-sense, and single-stranded RNA. ^1^ This virus recently caused an outbreak throughout the world, known as Covid-19, and poses a serious threat due to systemic inflammation and coagulopathy. ^1^ ^–^ ^3^ One known pathological inflammatory response triggered by Covid-19 infection is cytokine storm. ^2^ ^,^ ^3^ Cytokine storm is a fatal immune response characterized by unregulated activation of immune cells and massive generation of cytokines and chemical mediators. ^2^ ^,^ ^3^ Covid-19 infection can induce massive inflammatory response by rapidly activating pathogenic Th1 cells which produce proinflammatory cytokines, one of which is Interleukin-6 (IL-6). ^2^ ^,^ ^3^ And this IL-6 has been reported recently to have a strong predictor value of covid 19 severity. ^4^ ^–^ ^7^

Other common feature of Covid-19 is coagulopathy and thrombosis. ^1^ This coagulopathy is caused by endothelial dysfunction and activation of coagulation system which lead to thrombosis and microvascular occlusion, initially in the pulmonary vascular, and when the disease progresses, causing systemic thrombosis and organs failure. ^1^ ^,^ ^8^ ^–^ ^10^ IL-6 also play a role in amplifying the coagulation system by activating the epithelial cells, monocytes, and neutrophils, ^11^ which are supposed to inhibit anticoagulant protein S and antithrombin and enhance the activity of clotting factor VII, von Willebrand, and fibrinogen. A study by Gomez-Mesa, et al. stated that about 20%-50% of hospitalized patients with COVID-19 have elevated D-dimer, prolonged PT, thrombocytopenia, and/or low fibrinogen levels. ^1^ Therefore, in this study, we aim to seek the association between coagulopathy, which is measured by D-dimer, fibrinogen and PT level, along the Covid-19 progression. In this study, we also seek the IL-6 correlation with coagulation in Covid-19 to seek if IL-6 could have predictive value of Covid-19 associated Coagulopathy (CAC). ^12^

Ferritin level is reported elevated in recent studies and is associated with hemophagocytic lymphohistiocytosis/macrophage activation syndrome in Covid-19. ^5^ ^,^ ^13^ However, the absence of five out of eight characteristics in the previous Covid-19 study explains the differences between the disease. ^14^ Several retrospective studies have been conducted to observe the relationship between IL-6, PT, fibrinogen, and ferritin with Covid-19 progression. ^15^ ^–^ ^19^ In this prospective cohort study, we aimed to investigate the role of these parameters in Covid-19 disease progression.

According to studies of natural course of Covid-19, disease progression typically occurs within 7–14 days after the onset of symptoms. ^20^ Therefore, we use the cut-off within 14 days after symptoms to evaluate disease progression in this study and stop the observation afterward to make the sample homogenous.

Spo _2_/FiO _2_ is one component of the Covid-19 severity index and is simple, non-invasive, and readily available tool in most clinical settings in the world. ^21^ Spo _2_/FiO _2_ reflects directly the respiratory function. Spo _2_/FiO _2_ also reported by many studies to have predictive value of invasive mechanical ventilation ^22^ and mortality in Covid-19. ^21^ Thus, we also try to seek the correlation between IL-6, PT, fibrinogen, and ferritin with Spo _2_/FiO _2_ variables.

## Methods

### Study design

This was a prospective cohort study conducted from July 2020 to January 2021 at the Pertamina Central Hospital Modular Extension Simprug (Simprug Modular Extension Hospital, SMEH, Jakarta, Indonesia). Inclusion criteria were patients with Covid-19 confirmed by positive SARS-CoV-2 polymerase chain reaction (PCR) testing who were admitted from July 2020 to January 2021, aged 18 years or older; classified clinically moderate or severe Covid-19 (according to WHO interim guidance) ^23^; and willingness to provide blood sample. Exclusion criteria were history of chronic bleeding; prior anticoagulant therapy, undergoing hemodialysis; undergoing plasma convalescent clinical trial, or taking immunomodulation therapy (particularly IL-6 and intravenous immunoglobulin therapy) before or throughout the observation period.

### Sample collection and storage

First blood sample were drawn from patients who fulfilled the criteria, on the first day upon admission. The second blood sample were collected at the end of observation period which is on 14 ^th^ day upon admission. The collected blood samples were stored in the fridge at -20° Celsius at Clinical Pathology laboratory of Pertamina Central Hospital.

The blood samples were analyzed for IL-6, D-dimer, ferritin, fibrinogen, CRP, and prothrombin time. IL-6 was analyzed in the Integrated Laboratory of the Faculty of Medicine, Universitas Indonesia. During transferring IL-6 samples were maintained at constant temperature -20° Celsius according to Guidance on regulations for the Transport of Infectious Substances Guidance on regulations for the Transport of Infectious Substances 2007. IL-6 was analyzed using an ELISA IL6 Vmax microplate reader. Complete blood count, CRP, D-dimer, fibrinogen, and prothrombin time (PT) were analyzed using Sysmex CS2100i. Ferritin was analyzed using Cobas 601 Roche in the Clinical Pathology laboratory of Pertamina Central Hospital.

Other variables recorded in this study are non-invasive SpO _2_ and FiO _2_, which ratio is one parameter of Covid-19 disease severity index. ^21^ The data was recorded from a noninvasive pulse oximeter monitor. These variables are then correlated with the IL-6, ferritin, D-dimer, and PT level to seek the association. CRP level also recorded in this study and also analyzed to IL-6, D-dimer, ferritin, coagulopathy marker and SpO _2_/FiO _2_ ratio but not the main focus of the study.

### Assessment patients’ severity

The severity of the illness was classified according to the WHO interim guidance (published 13 ^th^ March 2020) ^23^ as mild, moderate, severe, or critical disease. Moderate case was defined as patient with clinical signs and symptoms of pneumonia but SpO _2_ levels is > 93% with room air. Severe cases was defined as patient with clinical signs and symptoms of pneumonia, and one of the following: 1) respiratory rate > 30 times per minute, 2) signs of respiratory distress, or SpO _2_ < 93% with room air. ^23^ ^,^ ^24^

Patients included in this study were patients who initially classified as moderate or severe disease. Patients were then observed up to day 14th or concluded earlier if the patients improved and discharged earlier, or worsened and passed away before day 14th. Worsening was defined as clinical disease severity progression from moderate to severe, or to critical disease or death. All patients received antibiotics, antiviral, corticosteroids, and anticoagulants (Fondaparinux, LMNH, or UFH) according to the standard hospital therapy based on WHO Covid-19 clinical guideline. ^23^ Detailed information regarding covid-19 therapy given is described in result section.

### Statistical analysis

All collected data were analyzed using Anaconda package program ^25^ and reported in the text, table, or figures. All *p-values* < 0.05 were considered statistically significant. Correlation test was used to analyze correlation between IL-6 with ferritin and coagulopathy variables (PT, D-dimer, and Fibrinogen). Method of cutoff points obtained using area under curve method. Cutoff points by receiver operator characteristic obtained by calculating the optimum sensitivity and specificity.

This study has been approved (approval date: June 29 ^th^ 2020) by the ethical committee, Universitas Indonesia (approval number: KET-650/UN2.F1/ETIK/PPM.00.02/2020), and Pertamina Central Hospital (approval number: 3315/B00000/2020-S8).

## Results

During the study period from July 2020 to January 2021, there were 374 moderate to severe Covid-19 patients treated at the SMEH. Of those, 117 patients were randomly selected, 42 were excluded due to incomplete data, one received convalescent plasma therapy, and 1 had lysis of blood samples. The remaining 73 patients were further analyzed.

The characteristics of the study subjects are summarized in Table 1. Of the 73 Covid-19 patients, 61 (84%) were classified as having severe Covid-19 and 12 (16%) were classified as having moderate Covid-19. The majority of the patients were male and the mean age of patients was 61 years (SD±12.74). Most of the patients were overweight, hospitalized at day 6 ^th^ of the illness with duration of 7 days. As the final observation outcomes, most of the subjects with moderate disease experienced improvement, and subjects with severe disease experienced worsening within 14 days of the illness. All subjects were given antiviral therapy either remdesivir, favipiravir, or oseltamivir based on clinical judgement and according to Indonesia National Guideline of Covid-19. ^24^ One patient was given oseltamivir because remdesivir or favipiravir was contraindicated (GFR <30 ml/min/1,73 m ^2^), and one patient was not in either antiviral because both remdesivir and favipiravir were contraindicated and oseltamivir was not available.

**Table 1.** Characteristics of the study subjects.

	Total (n=73)	Severe (n=61)	Moderate (n=12)
Age (years), mean (±SD)	61.00 (±12.74)	61.52 (±12.77)	56.50 (±12.24)
Gender			
Male (%)	47 (64%)	38 (62%)	9 (75%)
Female (%)	26 (36%)	23 (38%)	3 (25%)
Body Mass Index (kg/m ^2^), median (IQR)	26.28 (25.10–28.11)	26.17 (24.96–28.18)	27.01 (25.98–27.98)
Time from illness onset to admission (days), median (IQR)	6.00 (4.00–7.00)	6.00 (4.00–7.00)	6.00 (4.00–7.00)
Length of stay (days), median (IQR)	7 (5–8)	7 (5–8)	8 (7–11.75)
Final observation outcomes			
Improved (n)	37	26	11
Worsening (n)	36	35	1
Number of comorbidities			
None	22	20 (33%)	2 (17%)
One comorbidity	25	19 (31%)	6 (60%)
Two comorbidities	20	17 (28%)	3 (25%)
Three comorbidities	6	5 (8%)	1 (8%)
Therapy			
Oseltamivir (n)	1	1	0
Favipiravir (n)	29	23	6
Remdesivir (n)	42	36	6
Meropenem (n)	52	46	6
Levofloxacin (n)	35	31	4
Azithromycin (n)	31	29	2
Enoxaparin (n)	8	6	2
UFH (n)	47	43	4
Fondaparinux (n)	17	11	6
Methylprednisolone (n)	63	55	8
Dexamethasone (n)	8	5	3
Death	27	27	0
Survived	46	34	12
**First sample collection**			
D-dimer (mg/dL) ^a^, median (IQR)	1.21 (0.60–2.84)	1.45 (0.62–2.93)	0.61 (0.48–1.04)
Fibrinogen (mg/dL) ^b^, median (IQR)	678.00 (497.00–778.00)	703.00 (553.00–806.00)	469.50 (345.75–617.25)
Prothrombin time (seconds), median (IQR)	11.00 (10.00–11.00)	11.00 (10.00–11.00)	10.50 (10.00–11.00)
Ferritin (ng/mL) ^c^, median (IQR)	1505.00 (893.00–2334.00)	1505.00 (946.00–2334.00)	1433.00 (599.95–2058.00)
IL-6 (pg/mL), median (IQR)	8.00 (2.07–56.21)	8.00 (2.07–63.47)	9.07 (2.40–29.53)
*S*pO _2_/ *F*iO _2_ ratio, median (IQR)	170.18 (141.43–206.25)	148.48 (139.39–192.16)	395.24 (330.00–466.67)
**Second sample collection**			
D-dimer (mg/L) ^a^, median (IQR)	1.39 (0.85–3.86)	1.51 (1.03–4.31)	0.41 (0.32–0.81)
Fibrinogen (mg/dL) ^b^, median (IQR)	426.00 (342.00–597.00)	431.00 (352.00–611.00)	381.00 (327.25–480.50)
Prothrombin time (seconds), median (IQR)	11.00 (11.00–12.00)	11.00 (11.00–12.00)	10.50 (10.00–11.00)
Ferritin (ng/mL) ^c^, median (IQR)	1301.00 (813.00–2187.00)	1366.00 (933.00–2242.00)	1003.10 (361.75–1887.00)
IL-6 (pg/mL), median (IQR)	7.39 (1.50–33.26)	11.63 (2.45–36.54)	2.20 (0.82–4.50)
*S*pO _2_/ *F*iO _2_ ratio, median (IQR)	111.47 (94.00–394.64)	97.50 (93.97–247.50)	408.33 (408.33–411.46)

Notes: SD: Standard deviation, IQR: Interquartile range, n: number of samples, UFH: unfractionated heparin.

Normal lab reference range:

^a^ d-Dimer: <440 mg/L;

^b^ Fibrinogen: 200-400 mg/dL;

^c^ Ferritin: 20.00–500.00 ng/mL.

Patients suspected with secondary infection or sepsis were started empiric antibiotic(s) (either meropenem, levofloxacin, azithromycin, or combinations) and were changed later according to bacterial culture. Corticosteroid (dexamethasone 6 mg/day or methylprednisolone 32 mg/day for 10 days) was given to all severe cases, and selected moderate cases based on clinical judgement. Anticoagulants (unfractionated heparin, enoxaparin, or fondaparinux) were also given according to according to Indonesia National Guideline of Covid-19. ^24^ All patients not proven to have VTE, received thromboprophylaxis dose of anticoagulant, either UFH 2×5000 units subcutaneously, enoxaparin 1×4000 unit of anti-Xa (40 mg), or fondaparinux 1×2.5 mg. The dose of anticoagulant was adjusted based on clinical judgement and the increase of D-dimer level.

The variable distributions were analyzed according to disease severity and the worsening or improvement of patients’ condition, as shown in Table 2.

**Table 2.** Variables' distribution according to disease severity and outcomes.

	Severe		Moderate	
	Improved	Worsening	Improved	Worsening
Number of subjects	26	35	11	1
Age (years), median (IQR)	62.00 (47.00–71.75)	62.00 (55.00–69.00)	58.00 (50.50–64.00)	52.00 (52.00–52.00)
Body mass index (kg/m ^2^), median (IQR)	26.38 (25.17–28.19)	26.17 (24.85–27.85)	27.34 (26.18–28.03)	25.51 (25.51–25.51)
Day of worsening, median (IQR)	7.00 (6.00–7.75)	6.00 (5.00–7.50)	8.00 (7.00–10.00)	18.00 (18.00–18.00)
Time from illness onset to the worsening (days), median (IQR)	14 (13–17)	11 (10–14)	16 (12–18)	18
**First sample collection**				
D-dimer (mg/L), median (IQR)	1.34 (0.51–2.71)	1.50 (0.78–3.37)	0.63 (0.47–1.27)	0.60 (0.60–0.60)
Fibrinogen (mg/dL), median (IQR)	709.50 (515.50–799.00)	678.00 (568.00–808.50)	442.00 (344.50–554.00)	766.00 (766.00–766.00)
Prothrombin time (seconds), median (IQR)	11.00 (10.00–11.00)	11.00 (10.50–11.00)	11.00 (10.00–11.00)	10.00 (10.00–10.00)
Ferritin (ng/mL), median (IQR)	1137.00 (779.75–1799.00)	1906.00 (1361.50–2643.00)	1326.00 (533.70–1747.00)	2436.00 (2436.00–2436.00)
IL-6 (pg/mL), median (RIK)	6.27 (2.55–58.90)	10.78 (1.52–68.31)	12.19 (2.29–33.42)	3.93 (3.93–3.93)
*S*pO _2_/ *F*iO _2_, median (IQR)	179.41 (148.48–196.08)	143.94 (134.85–171.05)	333.33 (330.00–466.67)	457.14 (457.14–457.14)
**Second sample collection**				
D-dimer (mg/L), Median (IQR)	1.19 (0.94–1.64)	3.08 (1.33–9.63)	0.38 (0.32–0.55)	1.63 (1.63–1.63)
Fibrinogen (mg/dL), median (IQR)	406.00 (321.50–492.50)	498.00 (403.00–620.50)	361.00 (326.50–445.50)	491.00 (491.00–491.00)
Prothrombin Time (seconds), median (IQR)	11.00 (10.25–11.00)	11.00 (11.00–12.00)	10.00 (10.00–11.00)	11.00 (11.00–11.00)
Ferritin (ng/mL), median (IQR)	1030.00 (777.83–1452.25)	1938.00 (1154.00–2997.50)	719.20 (349.50–1743.00)	2163.00 (2163.00–2163.00)
IL-6 (pg/mL), median (IQR)	6.40 (1.47–17.21)	16.93 (3.58–94.38)	1.94 (0.64–3.75)	5.41 (5.41–5.41)
*S*pO _2_/ *F*iO _2_, Median (IQR)	247.50 (112.94–412.50)	95.00 (91.50–105.56)	408.33 (376.58–412.50)	267.57 (267.57–267.57)

Correlation coefficient analysis using the Spearman method showed p≥0.05 between IL-6 and ferritin, fibrinogen, D-dimer, and PT levels ( Table 3).

**Table 3.** Correlation between IL-6 and other variables.

IL-6 and	Correlation coefficient	p
Ferritin	0.08	0.50
D-dimer	−0.13	0.27
Fibrinogen	0.01	0.91
Prothrombin time	0.03	0.77

Spearman correlation.

**Figure 1.**
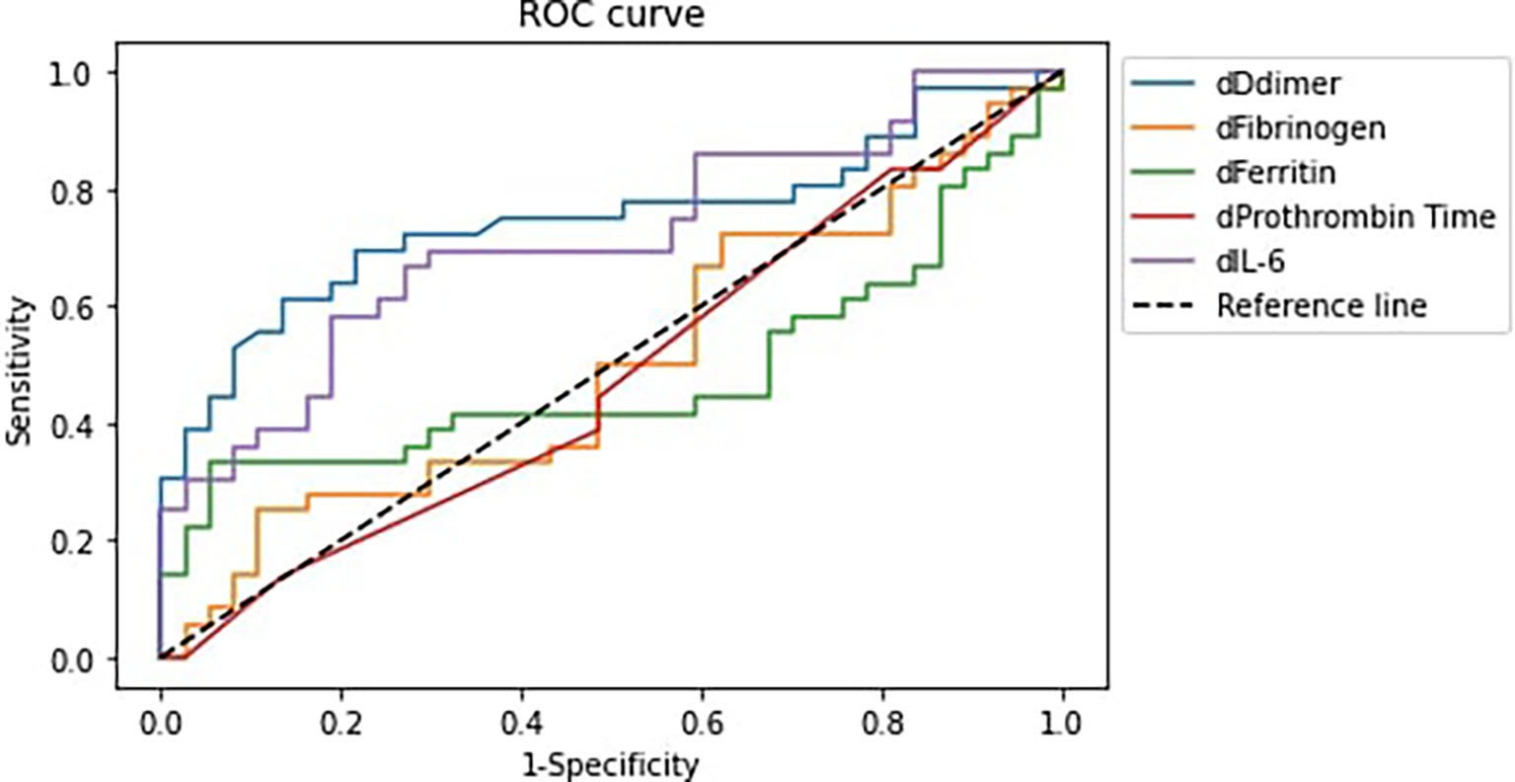
Receiver Operator Characteristic Curve of variable differences and Covid-19 patients’ deterioration. Notes: (1) dDdimer: D-dimer difference, dFibrinogen: Fibrinogen difference, dFerritin: Ferritin difference, dProthrombin Time: Prothrombin Time difference, dIL-6: IL-6 difference. (2) Difference is defined as the value at the end of observation – value at initial observation.

Figure 1 and Table 4 displayed the analysis of the receiver operating characteristic (ROC) curve (AUC) for correlation between elevated IL-6, ferritin, fibrinogen, D-dimer, and PT levels with Covid-19 patients’ deterioration.

**Table 4.** Area under ROC curve for the variable differences and Covid-19 patients’ deterioration.

Variables	AUC	95% CI
D-dimer	74.77%	63.48–86.07%
Fibrinogen	50.15%	36.81–63.49%
Prothrombin Time	47.67%	34.36–60.99%
Ferritin	48.42%	35.10–61.75%
IL-6	71.32%	59.48–83.17%

Notes: AUC: Area under the ROC curve, ROC: Receiver operator characteristic, CI: Confidence interval.

**Figure 2.**
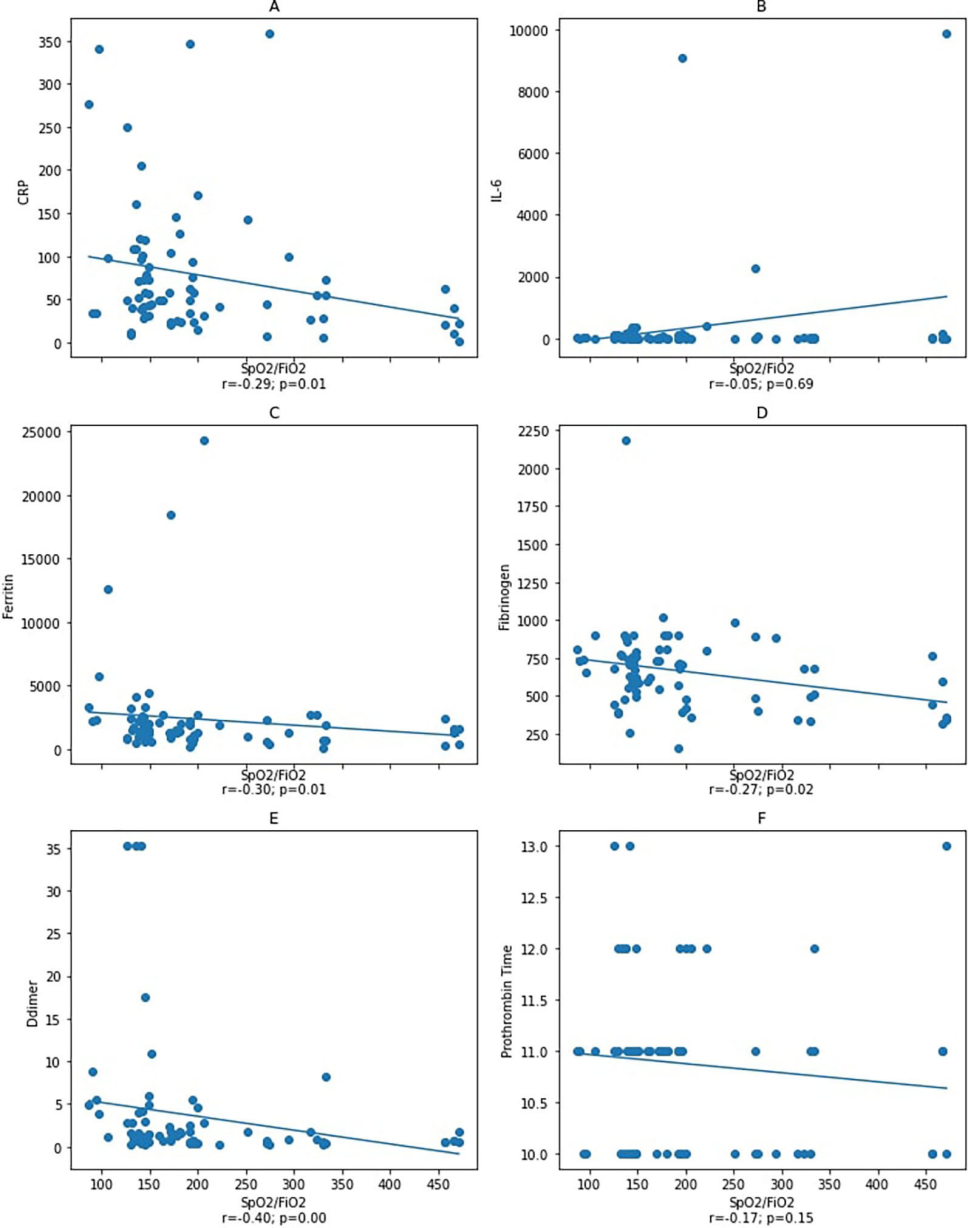
Correlation between IL-6, ferritin, D-dimer, and fibrinogen variables with *S*pO _2_/ *F*iO _2_ ratio at the initial observation. Spearman correlation test. CRP: C-reactive protein. Notes: A–D: correlation between *S*pO _2_/ *F*iO _2_ ratio and inflammatory markers CRP, IL-6, ferritin, and fibrinogen with a statistical significance was found between *S*pO _2_/ *F*iO _2_ ratio and inflammatory markers CRP, ferritin, and fibrinogen, but not with IL-6. Figure E and F: correlation between *S*pO _2_/ *F*iO _2_ ratio and coagulation markers D-dimer and prothrombin *T*ime with a statistical significance was found between *S*pO _2_/ *F*iO _2_ ratio and D-dimer, but not with prothrombin time.

**Figure 3.**
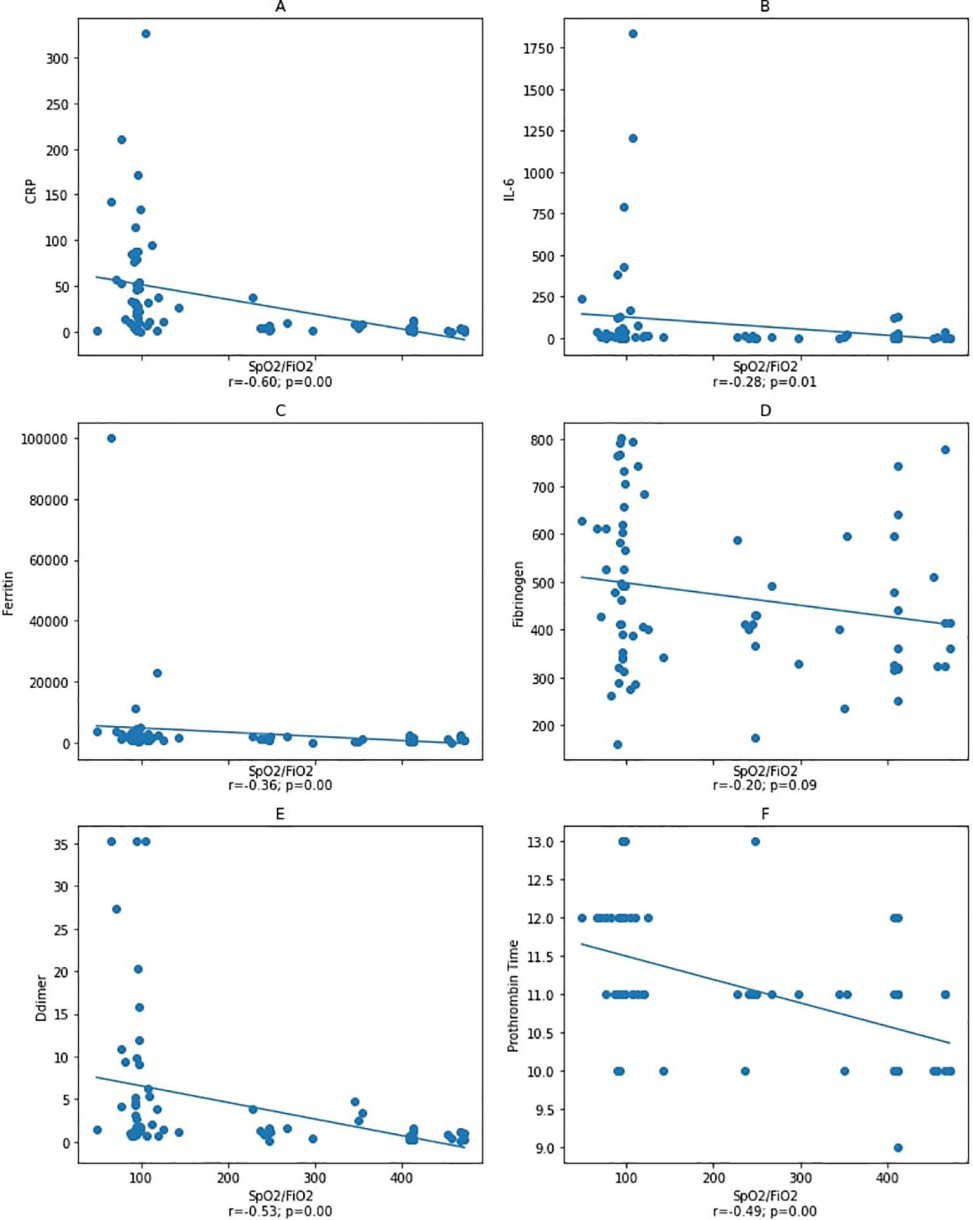
Correlation between IL-6, ferritin, D-dimer, and fibrinogen variables with *S*pO _2_/ *F*iO _2_ ratio at the end of observation. Spearman correlation test. CRP: C-reactive protein. Notes: A–D: correlation between *S*pO _2_/ *F*iO _2_ ratio and inflammatory markers CRP, IL-6, ferritin, and fibrinogen with a statistical significance was found between *S*pO _2_/ *F*iO _2_ ratio and inflammatory markers CRP and ferritin, but not with IL-6 and fibrinogen. Figure E and F: correlation between *S*pO _2_/ *F*iO _2_ ratio and coagulation markers with a statistical significance was found between *S*pO _2_/ *F*iO _2_ ratio and D-dimer and prothrombin time.

We found no correlation between IL-6 and other variables. Thus, as one of the disease severity index components, we aimed to investigate the correlation between the oxygen saturation ( *S*pO _2_)/fraction of inspired oxygen ( *F*iO _2_) ratio and IL-6, ferritin, fibrinogen, D-dimer, and PT ( Figures 2 and 3). As a result, our study demonstrated the correlation between IL-6, ferritin, fibrinogen, D-dimer, and PT and *S*pO _2_/ *F*iO _2_ ratio as the severity determinants.

## Discussion

### Characteristics of the study subjects

Males were predominant in this study (64%) with the age range 52–68 years old. Overall of the subjects were overweight. The median duration from illness onset to admission (O-A interval) was 6 days with median of length of stay (LoS) were 7 days in severe group and 8 days in moderate group.

The clinical course of the Covid-19 disease have been reported comprehensively, with the median O-A interval was 7 days, and illness onset to dyspnea interval was 8 days. ^26^ Subjects in this study had earlier median O-A interval which was 6 days. Peng *et al*. reported that the O-A interval less than 7 days predict the likelihood of disease progression in moderate-severe disease Covid 19. ^27^ But limitations were stated in aforementioned study that the health-seeking behavior of the patient and the domestic health system might influence the admission interval, aside from selection bias and confounding factors.

In this study, most subjects with moderate disease experienced improvement (11 subjects out of 12 [91%]) and most subjects with severe disease experienced worsening (35 subjects out of 61 [59%]). Almost half of severe group has no comorbidities, thus three fourth of the severe group had at least one comorbidity. While in moderate group, about 80% of subjects had at least one comorbidity. All of subjects in moderate group were survived, while in severe group, only 55% subjects survived despite therapies given. This study found that the comorbidity was not associated with the patient’s deterioration.

### Comparison of initial level of IL-6, ferritin, and coagulopathy marker with the level of the end of observation

Overall subjects came with increased initial D-dimer, fibrinogen, and ferritin levels. Those markers also higher in severe group compared to moderate group. This demonstrated the inflammation was higher and result in greater coagulopathy in severe disease. ^13^ ^,^ ^28^ ^–^ ^33^ Furthermore, they showed increased IL-6, ferritin, and fibrinogen reflecting the acute phase response (APR) of the disease. Subjects who visited the emergency department for more than 6 days (mean 7.5 days) had almost three times greater risk of worsening than those who came for less than 6 days (mean 4 days).

Levels of IL-6 at the first sample collection ( Table 1) were similar to the previous study. ^30^ In contrast to the studies by Jin Zhang *et al*. ^34^ and Awasthi *et al*., ^35^ in this study, subjects did not show a significant difference in IL-6 levels at the first measurement between moderate to severe illness. However, IL-6 level in worsening group had rising trend, while in contrast, IL-6 in improving group had down trend. This demonstrates the changes in IL-6 level might held more predictive value in disease progression than the initial value *per se.*

### Correlation between increased levels of IL-6 and ferritin

In acute phare response (APR), IL-6 is reported to be a pro-inflammatory cytokine that increase and determine infection-associated ferritin levels. ^32^ ^,^ ^36^ However, we did not found the aforementioned correlation between ferritin levels and IL-6 in the first and second sample collection ( Figure 3).

Although subjects did not show raised IL-6 level at admission, we found an increase in ferritin and fibrinogen levels, indicating that inflammation was great in initial state. Furthermore, other factors contributing to increased ferritin levels, such as epithelial damage, were also considered. Since a decrease in the *S*pO _2_/ *F*iO _2_ ratio was due to an acute respiratory distress syndrome (ARDS) manifestation, ^37^ ^,^ ^38^ the correlation between *S*pO _2_/ *F*iO _2_ ratio with ferritin and fibrinogen was investigated to determine whether the epithelial damage affected IL-6 and other inflammatory variables. Our result showed a statistically significant correlation between *S*pO _2_/ *F*iO _2_ ratio with ferritin and fibrinogen ( Figure 2). This correlation showed that inflammation affected the *S*pO _2_/ *F*iO _2_ ratio from the onset of the illness. However, since the increase of IL-6 was not correlated with *S*pO _2_/ *F*iO _2_ ratio, we assumed that other factors contributing to ferritin release in this study were greater than induced by IL-6. ^19^ ^,^ ^39^ According to a study by Zhi *et al*., several possibilities lead to the increase of ferritin: 1) pro-inflammatory cytokines stimuli ( *i.e.*, IL-1β, tumour necrosis factor α (TNF-α), and IL-6) caused an inflammatory reaction that damaged the cells, 2) intracellular ferritin leakage due to cell damage and inflammation, and 3) the ferritin leakage from the injured cells further triggers the damage of other cells *via* Fenton and Haber–Weiss’s reaction. ^13^

### Correlation between levels of IL-6 with D-dimer, fibrinogen, and PT

Inflammation will trigger the coagulation system characterized by changes in the value of D-dimer, fibrinogen, and PT at the time of viral entry. ^40^ Thus, we investigated the correlation between levels of IL-6 and markers of coagulopathy and inflammation.

Since there was no correlation found between IL-6, ferritin, and the incidence of coagulopathy, we concluded that coagulopathy in Covid-19 patients could occur disregard to IL-6 and ferritin levels. Such explanations for this finding are: 1) ARDS pathophysiology in the infected subjects with moderate and severe illness is related to a massive loss of the angiotensin-converting enzyme (ACE)-2 enzyme, causing damage to the alveolar epithelium and vascular endothelium, ^41^ 2) compartmentalization of the inflammatory cascade, as described by Chow and Tisoncik *et al*., ^42^ ^,^ ^43^ and 3) an acute phase response (APR) increase may be due to other pro-inflammatory cytokines, such as TNF-α or IL-1, ^31^ ^,^ ^39^ ^,^ ^40^ ^,^ ^44^ ^,^ ^45^ and the aforementioned vicious cycle of cellular destruction.

An analysis was carried out on the second blood collection after patients had a worsening during treatment to determine whether the incidence of coagulopathy correlates with IL-6 levels, in which the effect of IL-6 and cytokine storm was the utmost. The data from the second blood collection showed a significant correlation between the *S*pO _2_/ *F*iO _2_ ratio and the D-dimer, ferritin, PT, and IL-6 ( Figure 3). Furthermore, the D-dimer, fibrinogen, and PT were correlated with ferritin, while there was no correlation with IL-6. Our result demonstrated that inflammation correlates with coagulopathy, but conceivably not *via* a direct IL-6 pathway. To explain this finding, according to a study described by Sinha *et al*. that the interaction of mediators and pathways involved is not always constantly linear or uniform. ^46^

**Figure 4.**
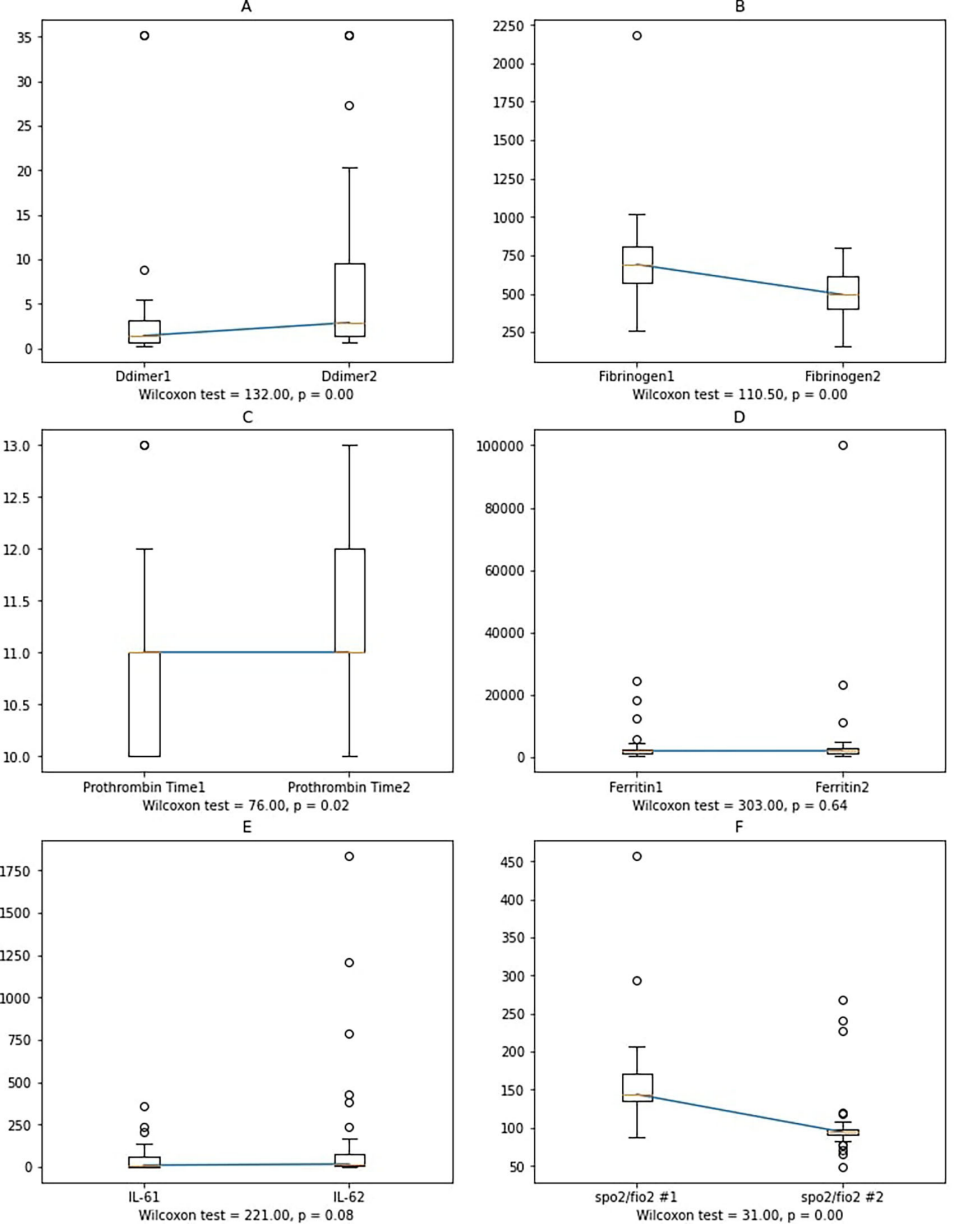
Variable alterations between the first and second sample collection in the worsening group. Notes: Ddimer1, Fibrinogen1, Prothrombin Time1, Ferritin1, IL-61: D-dimer, fibrinogen, prothrombin time, ferritin, and IL-6 at the first sample collection; Ddimer2, Fibrinogen2, Prothrombin Time2, Ferritin2, IL-62: D-Dimer, fibrinogen, prothrombin time, ferritin, and IL-6 at the second sample collection.

Worsening subjects showed significant changes in D-dimer, fibrinogen, and PT. Since there was no correlation between IL-6, ferritin and PT, fibrinogen, and D-Dimer, we determined the changes in the variables’ mean values between the first and second blood collections. Furthermore, Wilcoxon’s non-parametric test was performed since the data was not normally distributed ( Figure 4).

In worsening subjects, the second IL-6 blood collection had a higher value than the first one, albeit not statistically significant. Possible reasons include: 1) cytokines other than IL-6 affect the increase in D-dimer, fibrinogen, and ferritin levels, or 2) external factors, such as administration of anti-inflammatory corticosteroids and heparin, also have anti-inflammatory effects, ^47^ resulting in an insignificant increase of IL-6 and ferritin. During the study period, corticosteroids and heparin were administrated following the standard therapy for moderate and severe Covid-19 patients, or 3) large amounts of soluble IL-6 receptors production binds free IL-6 to reduce the concentration, as described by Garbers *et al*., ^48^ or 4) the presence of other cytokines (IL-6 cytokine family), including IL-11, ^49^ that also has a pro-inflammatory effect. ^50^

The disease severity was determined using WHO criteria for oxygen saturation, respiratory rate, and radiological findings. Most subjects were admitted to the emergency department with severe illness (83%), with a median *S*pO _2_/ *F*iO _2_ ratio was 97. In moderate illness, there were no differences in IL-6 at the baseline.

Our study found a negative correlation between D-dimer, fibrinogen, and PT levels with the SpO _2_/FiO _2_ ratio, reflecting the disease severity. This finding demonstrated that coagulopathy had a role in the patient’s deterioration even though SpO _2_/FiO _2_ ratio showed no correlation with IL-6. The worsening lung function was due to pre-existing inflammation before the increase in IL-6 may explain the correlation with the coagulation system.

Leisman ^45^ and Sinha ^46^ proposed that the pathophysiology of moderate and severe Covid-19 ARDS differs from typical ARDS, where the inflammatory system activation marked by increased IL-6 occurred only in a few Covid-19 ARDS patients. Our study showed that only 15.1% of subjects had IL-6 elevation at the initial examination, with an increase of 10-times the lower standard limit. Furthermore, if Covid-19 ARDS is considered a hyperinflammatory type, the IL-6 will be much lower than the value found in studies of the hyperinflammatory type that showed an increase of more than 100-times the lower standard limit.

The second blood collection sample showed that IL-6, ferritin, D-dimer, and PT had a statistically significant negative correlation with the *S*pO _2_/ *F*iO _2_ ratio, suggesting that deteriorating lung function is also correlated with inflammation and coagulopathy. However, we found no statistically significant correlation between IL-6 with D-dimer, fibrinogen, PT, and ferritin. As mentioned previously, this phenomenon may be caused by administering anti-inflammatory drugs that can reduce APR levels.

Fibrinogen is a soluble glycoprotein synthesized by the liver, and the activation can produce insoluble fibrin in the plasma. This process occurs *via* intrinsic and extrinsic pathways, and the activity is assessed by measuring PT.

Fibrinogen level is elevated in inflammatory conditions. ^39^ In patients who experienced worsening, we found that the fibrinogen levels at admission were higher than normal, with no changes in PT values. Thachil argued that the initial increase of fibrinogen would regulate inflammation. However, when the D-dimer levels continue to elevate with decreased fibrinogen levels, the protective role of fibrinogen ceases, and thrombus formation begins. ^51^ In our finding, the initial fibrinogen levels in moderate and severe Covid-19 were increased but decreased at the second blood collection sample analysis, which showed a tendency towards normal compared to the worsening group. This result demonstrated that hypercoagulable conditions might have a role in the pathology of the worsening group, a condition that differs from the hypothesis as reported by Thachil. ^51^ The hypercoagulable state can result from neutrophils’ subsequent pulmonary capillary endothelial activation, namely the formation of neutrophil extracellular traps (NETs). ^52^ ^–^ ^56^

### The correlation between ferritin levels with D-dimer, fibrinogen, and PT

As part of APRs, ferritin was negatively correlated with the *S*pO _2_/ *F*iO _2_ ratio ( Figure 2). We assumed that the inflammation process had occurred prior to the patients’ hospitalization, which explains the presence of a correlation between ferritin and the disease severity (moderate and severe). In the early phase, ferritin levels did not correlate with D-dimer, PT, and fibrinogen levels, which unlikely indicate that inflammation triggers coagulopathy.

On the initial observation, there was an elevation in ferritin levels in the subjects with moderate and severe illness ( Table 1). In the second blood collection sample, ferritin levels increased in the worsening but decreased in the improved group. Finally, we found the most significant changes in the improved group of moderate illness subjects ( Table 2), as also reported by Zhi *et al*. ^13^ To explain this finding, we hypothesized that 1) pro-inflammatory cytokines such as IL-1β, TNF-α, and IL-6 can increase ferritin synthesis, 2) damage to the cellular level due to inflammation will release intracellular ferritin, ^31^ and 3) in acidosis, increased production of reactive oxygen species induce the release of iron from the ferritin. In addition, Fenton and Haber–Weiss reaction increases the concentration of hydroxyl radicals and eventually damages the cells. Overall, this process will initiate a vicious cycle of cell damage by increasing ferritin concentration.

Rosario, ^57^ explained that ferritin has immunosuppressant and pro-inflammatory effects. The immunosuppressant properties were found in the ferritin H fraction, suppressing B lymphocyte antibodies’ production. Additionally, ferritin also reduces granulocytes’ phagocytosis, regulates the production of granulocytes-monocytes, and induces the production of IL-10 in lymphocytes. Moreover, it also inhibits the function of CXCR4 (chemokine receptor 4), an activator of the mitogen-activated protein kinase system that plays a role in the proliferation, differentiation, and migration of the cells. The role of ferritin in the inflammatory process has been described in hepatic stellate cells. For example, increased nuclear factor κB can promote the expression of inflammatory mediators, including IL-1β, and induce the production of nitric oxide synthase. This immunomodulatory process belongs to ferritin H and L properties. However, the role of ferritin as an inducer of inflammation or anti-inflammatory in Covid-19 infection has not been proven since the elevated ferritin type is still unknown. ^31^ ^,^ ^32^ ^,^ ^57^

The correlation between ferritin with fibrinogen, D-dimer, and PT in Covid-19 patients is recognized as a damage-associated molecular pattern that can modulate IL-6 concentrations. ^31^

There was no correlation between decreased *S*pO _2_/ *F*iO _2_ ratio and IL-6 in the first blood collection sample, but *S*pO _2_/ *F*iO _2_ ratio was correlated with acute-phase protein ferritin and fibrinogen. Albeit D-dimer, fibrinogen, and PT were correlated with the *S*pO _2_/ *F*iO _2_ ratio, we found no correlation between ferritin and those. Based on this finding, we demonstrated that the increase in APR does not correlate with the incidence of coagulopathy in Covid-19 since the condition has different mechanisms, including epithelial damage due to the entry of the SARS-CoV-2 virus and the increased production of NETs by neutrophils, as previously described.

### Alteration of IL-6, ferritin, fibrinogen, PT, and prediction of Covid-19 severity

Theoretically, inflammatory (IL-6 and ferritin) and coagulopathy (D-dimer, fibrinogen, and PT) markers are correlated with patients’ deterioration. ^4^ ^,^ ^5^ ^,^ ^13^ ^,^ ^58^ Interleukin-6 regulates the inflammatory process, and the elevation is associated with the worsening of Covid-19 patients. ^6^ ^,^ ^16^ In accordance with recent studies, this study also proved that increased levels of IL-6 can predict the deterioration of subjects.

Inflammation due to Covid-19 can activate epithelium, endothelium, macrophages, and neutrophils. Massive recruitment of neutrophils to the lungs and release of NETs regulates coagulation and IL-6 activation. ^54^ ^,^ ^59^ We found that alteration in D-dimer levels can predict the patients’ deterioration. Therefore, the availability of D-dimer in hospitals can justify its function as a routine examination.

Although the alteration of ferritin and fibrinogen cannot predict the patient’s deterioration, these APR markers can be elevated in inflammatory conditions. ^32^ ^,^ ^39^ Thus, administration of corticosteroid therapy or anti-inflammatory agents, such as heparin, can reduce the inflammation process, ^47^ ^,^ ^60^ as marked in our study by the unaltered ferritin and decreased fibrinogen levels ( Table 2).

Prothrombin time is the period required for plasma to clot after adding tissue factor. The reaction velocity is the result of coagulation factors activation that consist of coagulation factors XII to X. Furthermore, Yu Zhang *et al*. found that PT had a sensitivity of 83.54% and specificity of 65.22% for sepsis patients at the cut-off 20. ^61^ Our study showed a 75% quartile of 12 in severe Covid-19 subjects, describing that levels of PT were insensitive to measure patient’s deterioration. A similar finding was also reported in a retrospective study by Long *et al*., ^58^ where the initial measurement of PT was not correlated with the disease severity. This phenomenon may demonstrate the effect of hypercoagulation as the dominant pathophysiology in the early phase of the disease, in contrast to DIC in sepsis. ^62^ ^,^ ^63^

Our study limitation is that the IL-6’s diurnal variation cannot be eliminated since the use of a consecutive sampling method and the disease phase factor.

## Conclusion

In moderate and severe Covid-19 patients, there was a correlation between elevated IL-6 and D-dimer levels with disease deterioration. Groups with worsening outcome had higher presenting IL-6, Ferritin, D-dimer, and fibrinogen levels. IL-6 and D-dimer can be independent predictor for poor prognosis in Covid-19 disease progression, depicted by the upward trend of IL-6 and D-dimer level in worsened group. There was no correlation between elevated IL-6 levels with ferritin, D-dimer, fibrinogen, and PT levels.

## Informed consent statement

Written informed consent from the patient/patient’s family for the use and publication of the patient’s data was obtained from all subjects involved in the study. Informed consent was conceptualized according to local ethics committee, and hospital review committee.

## Data availability statement

### Underlying data

Underlying data cannot be shared due to privacy concerns. Data will be made available to readers and reviewers on request from Alvin Tagor Harahap ( alvinharahap@gmail.com).
