# Supplementary material for: The role of IL-6, ferritin, and coagulopathy in Covid-19 clinical progression
Source: F1000Res. 2023 Sep 22;11:1285. Originally published 2022 Nov 10. [Version 2] doi: 10.12688/f1000research.125115.2 (PMC10576189; doi:10.12688/f1000research.125115.2)
Supplement: Peer review history

# Reviewer report: Reviewer response for version 2

**DOI:** 10.5256/f1000research.156101.r208645

**Published:** 2023-10-13

**Authors:** Gopal Krishna

**Affiliations:** 1 Department of Neurosurgery, Pandit Bhagwat Dayal Sharma Post Graduate Institute of Medical Sciences, Rohtak, Haryana, India

The authors have revised the article satisfactorily.

Is the work clearly and accurately presented and does it cite the current literature?

Partly

If applicable, is the statistical analysis and its interpretation appropriate?

I cannot comment. A qualified statistician is required.

Are all the source data underlying the results available to ensure full reproducibility?

No

Is the study design appropriate and is the work technically sound?

Partly

Are the conclusions drawn adequately supported by the results?

Partly

Are sufficient details of methods and analysis provided to allow replication by others?

Partly

Reviewer Expertise:

Neuroendocrine disorders, Coagulation disorders in Neurosurgery, Neurospinal disorders, vascular Neurology

I confirm that I have read this submission and believe that I have an appropriate level of expertise to confirm that it is of an acceptable scientific standard.

# Reviewer report: Reviewer response for version 2

**DOI:** 10.5256/f1000research.156101.r208644

**Published:** 2023-10-06

**Authors:** Azlan Bin Husin

**Affiliations:** 1 Department of Internal Medicine, School of Medical Sciences, Universiti Sains Malaysia, Kubang Kerian, Malaysia

All previous comments have been addressed adequately

Is the work clearly and accurately presented and does it cite the current literature?

Partly

If applicable, is the statistical analysis and its interpretation appropriate?

I cannot comment. A qualified statistician is required.

Are all the source data underlying the results available to ensure full reproducibility?

Partly

Is the study design appropriate and is the work technically sound?

Partly

Are the conclusions drawn adequately supported by the results?

No

Are sufficient details of methods and analysis provided to allow replication by others?

No

Reviewer Expertise:

Clinical hematology, malignant hematology, benign hematology, autologous hematopoietic stem cell transplant

I confirm that I have read this submission and believe that I have an appropriate level of expertise to confirm that it is of an acceptable scientific standard.

# Reviewer report: Reviewer response for version 1

**DOI:** 10.5256/f1000research.137385.r168232

**Published:** 2023-05-22

**Authors:** Gopal Krishna

**Affiliations:** 1 Department of Neurosurgery, Pandit Bhagwat Dayal Sharma Post Graduate Institute of Medical Sciences, Rohtak, Haryana, India

The authors have conducted a good study. However, the parts of article are not properly organized. There is extensive overlap of results and discussion. The write up needs extensive revision. Some important aspects to be addressed:

**Introduction:**

1. The introduction is very brief. Basic concepts regarding pathophysiological aspects of inflammation in COVID-19 may be described here. The role of parameters included in metadata and their behaviour in COVID-19 may be elucidated. 
2. The treatment protocol pertaining to corticosteroids, low molecular weight heparin or UFH, and antiviral medications e.g. remdesivir etc. should be mentioned. And the modifications of these drugs according to the severity of disease.

**Methods **

1. The exclusion criteria should explain whether patients on oral anticoagulants, liver dysfunction were included or not. As a large size of study population had diabetes and hypertension, underlying coronary artery disease is likely to be present. Such population may have been on oral anticoagulant therapy prior to COVID-19.
2. C-reactive protein have been also studied and detailed in result section. It is a new parameter which is described by authors in results bearing no mention in methods.
3. The criteria of worsening of patients need to be described well according to citation used or modification, if any.

**Results:**

1. "The patients were randomized" is not correct as selection of patients and their classification was the decision of authors.
2. The part of discussion, "Fig 4.Variable alterations between the first and second sample collection in the worsening group" should be explained in the result section.
3. With using terms like "most" and "majority", the percentages must be mentioned next to them.
4. Repetition of many result parameters in discussion e.g., "males were most commonly affected in this study" is not appropriate.
5. Attempt may be made to include data on patients who worsened.
6. The normal reference laboratory values should be given for all parameters used in the study.

**Discussion:**

1. This section needs to be rewritten as there are many grammatical errors. The authors must seek professional help.
2. Many lines and paragraphs are not correct. e.g., ”This study found that increased levels of IL-6 can predict the deterioration of subjects, but it is insufficient to justify **it as a routine examination**. Additionally, **uncorrelated IL-6 with coagulopathy markers**, perhaps due to the ability of IL-6 to activate the inflammatory cascade in multiple body systems, ^32^ inducing amplification of the cascade".
3. The part, “Although subjects did not experience an inflammatory process at admission" is not acceptable. Subjects do not experience inflammatory process they suffer with symptoms.
4. In subheading "Correlation between levels of IL-6 with D-dimer, fibrinogen, and PT", "Since there was no correlation found between IL-6, ferritin, and the incidence of coagulopathy, we concluded. The word "concluded" may be changed to "assumed". Authors should mention e.g., "in the first sample analysis..." and then start elaboration. The entire part ****does not fit well with subsequent portions of ****discussion.  Authors are advised to write in flow oriented manner.
5. The authors are also advised to discuss the results and its implications with a statistician. 

Is the work clearly and accurately presented and does it cite the current literature?

Partly

If applicable, is the statistical analysis and its interpretation appropriate?

I cannot comment. A qualified statistician is required.

Are all the source data underlying the results available to ensure full reproducibility?

No

Is the study design appropriate and is the work technically sound?

Partly

Are the conclusions drawn adequately supported by the results?

Partly

Are sufficient details of methods and analysis provided to allow replication by others?

Partly

Reviewer Expertise:

Neuroendocrine disorders, Coagulation disorders in Neurosurgery, Neurospinal disorders, vascular Neurology

I confirm that I have read this submission and believe that I have an appropriate level of expertise to confirm that it is of an acceptable scientific standard, however I have significant reservations, as outlined above.

# Response

**Published:** 2023-09-15

**Authors:** Cosphiadi Irawan

**Affiliations:** Hematology and Oncology Medici, University of Indonesia, Depok, West Java, Indonesia

Thank you for your constructive review. We have made some corrections in respond to your feedback.

**Introduction**

 1. The introduction is very brief. Basic concepts regarding pathophysiological aspects of inflammation in COVID-19 may be described here. The role of parameters included in metadata and their behaviour in COVID-19 may be elucidated. 

- We have reconstructed the introduction part to be more comprehensive and narrative. We included the concept of pathophysiological aspect of inflammation in Covid 19 and the reason we did the study

2. The treatment protocol pertaining to corticosteroids, low molecular weight heparin or UFH, and antiviral medications e.g. remdesivir etc. should be mentioned. And the modifications of these drugs according to the severity of disease. 

- We have added the protocol of covid 19 management used in this study

**Methods**

 1. The exclusion criteria should explain whether patients on oral anticoagulants, liver dysfunction were included or not. As a large size of study population had diabetes and hypertension, underlying coronary artery disease is likely to be present. Such population may have been on oral anticoagulant therapy prior to COVID-19

- We have added more detailed criteria of exclusion in the methods section and corrected ambiguous statement in the methods. The population with prior anticoagulant therapy was excluded

2. C-reactive protein have been also studied and detailed in result section. It is a new parameter which is described by authors in results bearing no mention in methods  

- We have added explanation of CRP analysis in this study in method section. CRP was not the main focus of this study thus we did not describe CRP comprehensively in the method section.

3. "The patients were randomized" is not correct as selection of patients and their classification was the decision of authors.

- we agreed and have revised the grammar

4. The part of discussion, "Fig 4.Variable alterations between the first and second sample collection in the worsening group" should be explained in the result section.

- We added more explanations about fig 4

5. With using terms like "most" and "majority", the percentages must be mentioned next to them.

- We agreed and added percentages next to them

6. Repetition of many result parameters in discussion e.g., "males were most commonly affected in this study" is not appropriate.

- we reconstructed the statements and reduced repetitions

7. Attempt may be made to include data on patients who worsened.

- we added some narratives about worsened patients in discussion

8. The normal reference laboratory values should be given for all parameters used in the study.

- We added normal reference laboratory values as footnotes below the table 

**Discussion:**

 1. This section needs to be rewritten as there are many grammatical errors. The authors must seek professional help.

- We already consulted with professional translator and we made corrections in discussion. We hope the corrected version will convey the study result more clearly

2. Many lines and paragraphs are not correct. e.g., ”This study found that increased levels of IL-6 can predict the deterioration of subjects, but it is insufficient to justify **it as a routine examination**. Additionally, **uncorrelated IL-6 with coagulopathy markers**s due to the ability of IL-6 to activate the inflammatory cascade in multiple body systems, ^32^ inducing amplification of the cascade".,

- we reconstructed the statements

3. The part, “Although subjects did not experience an inflammatory process at admission" is not acceptable. Subjects do not experience inflammatory process they suffer with symptoms.

- we reconstructed the statement

4. In subheading "Correlation between levels of IL-6 with D-dimer, fibrinogen, and PT", "Since there was no correlation found between IL-6, ferritin, and the incidence of coagulopathy, we concluded. The word "concluded" may be changed to "assumed". Authors should mention e.g., "in the first sample analysis..." and then start elaboration. The entire part ****does not fit well with subsequent portions of ****discussion.  Authors are advised to write in flow oriented manner.

- we tried to reconstruct the structure and add more conjunctive narrations. 

5. The authors are also advised to discuss the results and its implications with a statistician.

# Reviewer report: Reviewer response for version 1

**DOI:** 10.5256/f1000research.137385.r164119

**Published:** 2023-03-13

**Authors:** Azlan Bin Husin

**Affiliations:** 1 Department of Internal Medicine, School of Medical Sciences, Universiti Sains Malaysia, Kubang Kerian, Malaysia

Overall writing of this manuscript requires extensive language and grammatical improvement. Subject matter of interest were properly introduced but some of methodology parts were not defined. Hence, there is a missing connection between the subject of interest, methodology, results and discussion.

**Introduction**

 The introduction needs to be revised to better contextualize on the work performed.

1. Provide rationale of evaluating only prothrombin time and D-dimer instead of adding partial thromboplastin time too whereas it was mentioned in introduction that von Willebrand activity also enhanced.
2. The term "effectiveness of parameters" in Introduction, para 2 is inappropriate.
3. Provide rationale of evaluating SpO2/FiO2 ratio as the chosen severity index to compared with selected inflammatory markers.

**Methods**

 This section requires substantial revision and improvement 

1. Define the term "BALF" in Study design. 
2. Clarify what happen if enrolled patients received IL-6 inhibitor or Ig later part of hospital stay? Example before day 14. Were they withdrawn or included in the analysis?
3. CRP was not mentioned in the sample collection and tables but is showed in the graphs.
4. Provide rationale and appropriate reference of using cut off point 14 days for assessment in the Assessment of patient's severity. What if patient stay longer than that and changed in clinical status?
5. Revise the term "or considered complete" used in Assessment of patient's severity. Perhaps use 'censored'?
6. Provide reference and appropriate citations used for definition of COVID-19 disease severity progression or worsening.
7. Provide more information on the standardized approach of administering antibiotics, antivirals, corticosteroids and anticoagulant in the study center. This is to understand the different types of these agents used and possible interaction with the variables assessed.
8. The term "coagulopathy" used in Statistical analysis, para 1 should be defined earlier.
9. The term "randomized" in Results, para 1 is incorrect because the study population was not randomized. Perhaps 'randomly selected' is more suitable?

**Results**

1. For Table 1; should add normal reference range as foot note.
2. Statement "subjects with severe disease experienced worsening on day 14..." should be "subjects with severe disease experienced worsening within 14 days of..."
3. Percentage of hypertension should be reflected in the table 1; can be as footnote.
4. The statement "All subjects were given the combination of remdesivir, favipiravir, and oseltamivir" is not consistent with the actual number of patients received it.
5. Author should describe the approach or basis of how different types of antibiotics given to these patients. This important to understand factors may influence the inflammatory markers evaluated in different category of patients.
6. Statement "Correlation coefficient analysis using the Spearman method showed p≥0.05 between IL-6 and ferritin, fibrinogen, D-dimer, and PT" should be, "Correlation coefficient analysis using the Spearman method showed p≥0.05 between IL-6 and ferritin, fibrinogen, D-dimer, and PT levels".
7. Disease severity index was not mentioned in introduction and methodology.

**Discussion;** *Characteristics of the study subjects*

1. Para 3, line 2: statement "In this phase, most patients begin to feel shortness of breath accompanied by hypoxia" is this refers to all mild cases? If so, then it is incorrect statement.

**Discussion:** *Correlation between increased levels of IL-6 and ferritin*

1. Para 2, line 1: statement should be "...patient did not show raised inflammatory markers...".
2. The argument using Zhi *et al., *findings was rather contradicting to author's finding of no correlation with IL-6.

**Discussion:** *Correlation between levels of IL-6 with D-dimer, fibrinogen, and PT*

1. It is difficult to analyze and to explain what really happened among worsening patients as no clear definition of this event and what type of treatment they received?

**Conclusion**

1. Perhaps should include differences in inflammatory and hemostatic response between initial presentation and as patient became worse.

Is the work clearly and accurately presented and does it cite the current literature?

Partly

If applicable, is the statistical analysis and its interpretation appropriate?

I cannot comment. A qualified statistician is required.

Are all the source data underlying the results available to ensure full reproducibility?

Partly

Is the study design appropriate and is the work technically sound?

Partly

Are the conclusions drawn adequately supported by the results?

No

Are sufficient details of methods and analysis provided to allow replication by others?

No

Reviewer Expertise:

Clinical hematology, malignant hematology, benign hematology, autologous hematopoietic stem cell transplant

I confirm that I have read this submission and believe that I have an appropriate level of expertise to state that I do not consider it to be of an acceptable scientific standard, for reasons outlined above.

# Response

**Published:** 2023-09-17

**Authors:** Cosphiadi Irawan

**Affiliations:** Hematology and Oncology Medici, University of Indonesia, Depok, West Java, Indonesia

**Dear Azlan Bin Husin**

**We thank you for your constructive feedback. We made revisions in our article and hope you would not mind to review once again our article if it is eligible for indexing.**

**Introduction**

 The introduction needs to be revised to better contextualize on the work performed.

 1. Provide rationale of evaluating only prothrombin time and D-dimer instead of adding partial thromboplastin time too whereas it was mentioned in introduction that von Willebrand activity also enhanced.

- we added explanations in the introduction

2. The term "effectiveness of parameters" in Introduction, para 2 is inappropriate.

- we fixed the grammar

3. Provide rationale of evaluating SpO2/FiO2 ratio as the chosen severity index to compared with selected inflammatory markers.

- we added explanations in the introduction

**Methods**

 This section requires substantial revision and improvement 

 1. Define the term "BALF" in Study design. 

- we added some explanations

2. Clarify what happen if enrolled patients received IL-6 inhibitor or Ig later part of hospital stay? Example before day 14. Were they withdrawn or included in the analysis?

- the subjects were exempted. We revised the statement regarding the exclusion criteria

3. CRP was not mentioned in the sample collection and tables but is showed in the graphs.

- We added some explanations in sample collection but we 

4. Provide rationale and appropriate reference of using cut off point 14 days for assessment in the Assessment of patient's severity. What if patient stay longer than that and changed in clinical status?

- We used 14 days as cutoff based on studies reported the mean duration of disease progression typically occur at 7-14 days after onset of symptoms

Revise the term "or considered complete" used in Assessment of patient's severity. Perhaps use 'censored'?

- We agreed with your feedback. we fixed the sentence

Provide reference and appropriate citations used for definition of COVID-19 disease severity progression or worsening.

- We added references and explanations regarding Covid-19 disease severity

Provide more information on the standardized approach of administering antibiotics, antivirals, corticosteroids and anticoagulant in the study center. This is to understand the different types of these agents used and possible interaction with the variables assessed.

- We added explanations of management protocol used in our center, but mostly were based on clinical judgement

The term "coagulopathy" used in Statistical analysis, para 1 should be defined earlier.

- We added definition of coagulopathy in introduction

The term "randomized" in Results, para 1 is incorrect because the study population was not randomized. Perhaps 'randomly selected' is more suitable?

- We revised the sentence like you suggested

**Results**

 For Table 1; should add normal reference range as foot note.

- We added normal reference in table footnote

Statement "subjects with severe disease experienced worsening on day 14..." should be "subjects with severe disease experienced worsening within 14 days of..."

- We revised the statement

Percentage of hypertension should be reflected in the table 1; can be as footnote.

- We decided to not mention any comorbidities percentage

The statement "All subjects were given the combination of remdesivir, favipiravir, and oseltamivir" is not consistent with the actual number of patients received it.

- We revised the statements

Author should describe the approach or basis of how different types of antibiotics given to these patients. This important to understand factors may influence the inflammatory markers evaluated in different category of patients.

- We added explanations in results section regarding the selection of type of antibiotics given to the patients

Statement "Correlation coefficient analysis using the Spearman method showed p≥0.05 between IL-6 and ferritin, fibrinogen, D-dimer, and PT" should be, "Correlation coefficient analysis using the Spearman method showed p≥0.05 between IL-6 and ferritin, fibrinogen, D-dimer, and PT levels".

- We revised the sentence like you suggested

Disease severity index was not mentioned in introduction and methodology.

- We added explanations regarding disease severity index in introduction

**Discussion;** *Characteristics of the study subjects*

 Para 3, line 2: statement "In this phase, most patients begin to feel shortness of breath accompanied by hypoxia" is this refers to all mild cases? If so, then it is incorrect statement.

- We revised the statement. What we meant was most patients begin to feel chest discomfort and in moderate or severe cases, begin to feel shortness of breath.

**Discussion:** *Correlation between increased levels of IL-6 and ferritin*

 Para 2, line 1: statement should be "...patient did not show raised inflammatory markers...".

 The argument using Zhi *et al., *findings was rather contradicting to author's finding of no correlation with IL-6.

- We reconstructed the statements.

**Discussion:** *Correlation between levels of IL-6 with D-dimer, fibrinogen, and PT*

 It is difficult to analyze and to explain what really happened among worsening patients as no clear definition of this event and what type of treatment they received?

- We tried to explain the management protocol used in the result section and explain what happened in worsening group based on the markers analysed

**Conclusion**

 Perhaps should include differences in inflammatory and hemostatic response between initial presentation and as patient became worse

- We added more explanations regarding the difference in inflammatory and coagulation marker between initial and later sample.
